# Temperature Effects on the Crystalline Structure of iPP Containing Different Solvent-Treated TMB-5 Nucleating Agents

**DOI:** 10.3390/polym15030514

**Published:** 2023-01-18

**Authors:** Baojing Luo, Sheng Xu, Jing Yang, Qing Zhang, Jing Yu, Lihua Liu, Xiangjun Meng

**Affiliations:** 1 Department of Chemistry, Tangshan Normal University, Tangshan 063000, China; 2 Hebei Provincial Key Laboratory of Degradable Polymers, Tangshan Normal University, Tangshan 063000, China; 3 Department of Software and Communication, Tianjin Sino-German University of Applied Sciences, Tianjin 300072, China

**Keywords:** iPP, nucleating agent, crystalline structure, TMB-5, temperature

## Abstract

TMB-5 nucleating agent (NA) treated by different solvents were used as the *β*-NA of iPP. The effects of temperature on the crystalline structure of different iPP/TMB-5, as well as the crystallization and melting behaviors were investigated. It was found that strong polar solvent treated TMB-5 (TMB-5_DMSO_ and TMB-5_DMF_) could induce more *β*-crystal at high T_c_ = 140 °C than the other TMB-5 NAs, while the *β*-crystal inducing efficiency of untreated TMB-5 (TMB-5_UT_) and non-polar solvent treated TMB-5 (TMB-5_LP_) is seriously reduced at high T_c_ = 140 °C. TMB-5_DMSO_ can induce a high and stable content of *β*-crystal with K*_β_* = 83–94% within T_c_ = 90–140 °C, and TMB-5_ODCB_ can induce a high content of *β*-crystal with K*_β_* > 91.3% within T_c_ = 90–130 °C. TMB-5_DMF_ is the most temperature-sensitive one, but can induce a high fraction of *β*-crystal with K*_β_* > 92% both at low T_c_ = 90 °C and high T_c_ = 140 °C. High temperature pre-crystallization at T_pc_ = 150 °C tremendously reduces the *β*-crystal inducing efficiency of all TMB-5 NAs. TMB-5_UT_ and TMB-5_LP_ exhibit higher nucleating efficiency than TMB-5_DMSO_, TMB-5_DMF_ and TMB-5_ODCB_. During the non-isothermal crystallization process, TMB-5_UT_ induced *β*-crystal possesses higher structural perfection and stability, while TMB-5_LP_ is more likely to induce *α*-crystal with considerable quantity and stability. The structural perfection and stability of TMB-5 induced *β*-crystal can be enhanced with appropriate increasing of T_c_.

## 1. Introduction

The polymorphic phenomenon is commonly found in semicrystalline polymers resulted from the different conformations and/or packing modes of molecular chains in the crystal cells [1]. The final crystalline modifications and crystalline structures depend on both the structure of molecular chains and the external crystallization conditions (e.g., the thermal conditions, the mechanical conditions, and the addition of nucleating agent, etc.). The macroscopic performances of polymer materials depend on their inner structures, thus, to tailor the crystalline structures of semicrystalline polymer materials is beneficial to expand its industrial applications.

Isotactic polypropylene (iPP) is a semicrystalline polymer which exhibits different crystalline modifications, such as monoclinic *α*-form, trigonal *β*-form, orthorhombic *γ*-form, smectic and *ε*-form [2]. As known, *α*-iPP is the thermodynamically most stable phase among all crystalline modifications of iPP, which is obtained under common melting processing conditions [3,4]. The unique structure endows *β*-iPP crystals with special performances [5]. The main characteristic of *β*-iPP, apart from its crystalline structure, is its melting temperature, that is, the *β*-iPP crystals melt at a lower temperature (~154 °C) compared with the *α*-iPP crystals (~165 °C) and can recrystallized into *α*-iPP after melting [6].

Due to the better toughness, improved heat resistance [1], high impact and tear strength [7] and improved elongation at break [8], *β*-iPP has attracted much attention in both academic and industrial research. Some researchers found that the Izod impact strength, flexural modulus and heat distortion temperature of an injection molded polypropylene increased with the *β*-crystal content within a certain range [9]. The thermodynamically metastable *β*-iPP crystals can only be obtained by certain specific nucleating agents (NAs) [7,10], in sheared iPP melt, with crystallization in a temperature gradient, or by quenching the iPP melt rapidly to 100–130 °C [2]. Among all methods, the introduction of selective *β*-NA is the most reliable method to produce iPP samples rich in *β*-modification [10,11,12]. The first reported efficient *β*-NA was *γ*-quinacridone that could dramatically enhance the relative fraction of the *β*-crystal (K*_β_* value) in iPP even at an extremely low content range [13,14]. Some other NAs such as calcium salts of suberic acid and pimelic acid, 2,3-dimethylcyclohexyl substituted 1,3,5-benzenetrisamide, *N*,*N’*-dicyclohexyl-terephthalamide, *N*,*N’*-dicyclohexyl-2,6-naphthalenedicarboxamide (NJS) are also the most studied *β*-NAs for iPP [2].

The efficiency of *β*-NAs depends heavily on the concentration, dispersion and assemble morphology of the NAs, as well as the thermal history [12,15,16,17]. The growth rate of *β*-iPP crystal is higher than that of *α*-iPP crystal in a special temperature range about 100–140 °C [8,18,19]. With a temperature above 140 °C or below 100 °C, the *β*-to-*α* growth transition takes place which hinders the formation of *β*-iPP crystal. Dong et al. [8] investigated the crystallization behavior and morphological development of iPP with an aryl amide derivative (TMB-5) as *β*-NA of iPP, and found the *β*-crystal content remarkably increases above a critical NA concentration (0.05 %) and obviously decreases at higher crystallization temperatures T_c_ = 140 °C, 150 °C. Intelligent works of Li et al. [17] showed that high processing temperature promoted the dissolution of TMB-5 in iPP melt, and the dissolved NA self-assemble into high aspect ratio fibrils which induced the formation of anisotropic iPP crystals rich in *β*-form. Luo et al. [12] investigated the coupling effects of toughening modification and solid die drawing process on the morphology and mechanical properties of PP/TMB-5 composites with POE, and found that the *β*-*α* transformation occurred when applied a die drawing consequently with a huge reduction in *β*-crystal. Kang et al. investigated the crystallization behavior and polymorphic compositions of TMB-5 modified iPP/multi-walled carbon nanotubes composites, and found that higher cooling rate encourages the formation of higher proportion of *β*-phase with higher thermal stability, and the end temperature of cooling T_end_ = 100 °C can eliminate the *β*-*α* recrystallization during the subsequent heating enhancing the thermal stability of the *β*-phase [20].

TMB-5 is a commercialized *β*-form NA for iPP which has been widely used to study the crystallization behavior and crystalline structure of *β*-nucleated iPP [21]. It is the first choice for aromatic diamide *β*-NA, and also the earliest industrial application of a class of *β*-NA [22]. He et al. investigated the dependence of *β*-crystal formation of iPP on crystallization conditions, and found that the *β*-crystal fraction of TMB-5 nucleated iPP decreases with increasing cooling rate until a stable value [22]. The results of another investigation on the dynamic crystallization and melting behavior of *β*-nucleated iPP with different stereo-defect distribution also indicated that slow cooling rate favors the formation of high *β*-fraction, furthermore, high crystallization temperature favors the crystallization of *α*-phase accelerated by TMB-5 due to the dual-selectivity of TMB-5 NA [23]. However, Dong et al. found that fast cooling is favorable for the formation of *β*-iPP crystal [24]. TMB-5 has good thermal stability and heat resistance, and also has a good compatibility in iPP melt [24,25]. It has been proved that TMB-5 dissolved partially or completely in the iPP melt and recrystallized during the subsequent cooling process [8,26]. The dissolution and self-assembled morphology depend on the concentration of TMB-5 [26] and the final heating temperature [21]. The self-assembly morphology of TMB-5 has great influence on the crystalline structure and morphology of iPP. Until now, the influence of TMB-5 self-assembly morphology on the crystallization of iPP has mostly focused on the NA concentration, heating temperature, rising and cooling rates. With increasing the TMB-5 concentration, the assembled morphologies of TMB-5 in iPP melt change from dispersed nanoparticle, fibril, and needle structure with increasing TMB-5 concentration, among which the fibril and needle assembled TMB-5 induced more formation of *β*-iPP crystal [26]. Supercritical concentration of TMB-5 leads to a partial dissolution of TMB-5 particles in iPP melt or even a phase separation [26]. It was also found that TMB-5 almost completely dissolved in iPP melt when heated to 250 °C and then recrystallized from iPP melt in the form of very fine needle crystals [21]. 

Among the literatures, few studies have been focused on the influence of the solvent-treated TMB-5 NA on the crystalline structures and crystallization behaviors of iPP. In present work, different solvent-treated TMB-5 NAs with different initial particle morphologies were used as the *β*-NA of iPP. The main objective of this article is to investigate the effects of crystallization temperature on the *β*-iPP inducing efficiency of the different solvent-treated TMB-5, as well as the crystallization behaviors and crystalline structures of iPP.

## 2. Materials and Methods

### 2.1. Materials

IPP (trade name T1701, Mw = 3.0 × 10^5^ g/mol, Mn = 7.4 × 10^4^ g/mol and the melting temperature 167.7 °C) used in this study was provided by Yanshan Petrochemical Corp. Inc. (Beijing, China).

An aryl amide-based compound (trade name TMB-5) was used as the *β*-NA of iPP. TMB-5 has a similar chemical structure to *N*,*N*’-dicyclohexyl-2,6-naphthalenedicarboxamide. The raw TMB-5 powder was supplied by Chemical Institute of Shanxi, and the solvent-treated TMB-5 NAs which were, respectively, heated, cooled and filtered in dimethylsulfoxide (DMSO), dimethyl formamide (DMF), ortho-dichlorobenzene (ODCB) and liquid paraffin (LP), were kindly provided by School of Chemical Engineering, Tianjin University. For the sake of description, the solvent-treated TMB-5 NAs are designated as TMB-5_DMSO_, TMB-5_DMF_, TMB-5_ODCB_ and TMB-5_LP_, and the untreated raw TMB-5 is named as TMB-5_UT_. The optical microscopic pictures of the five TMB-5 NAs are provided in Figure S1, exhibiting their different initial particle morphologies.

### 2.2. Sample Preparation

The iPP granules were, respectively, melt-blended with 0.1 wt% and 0.05 wt% TMB-5 NAs (TMB-5_UT_, TMB-5_DMSO_, TMB-5_DMF_, TMB-5_ODCB_ and TMB-5_LP_) at 190 °C for 10 min via the 60 mL chamber of XSS-300 torque rheometer with a rotation speed of 30 r/min. The TMB-5 modified iPP samples are named as iPP/xTMB-5_y_, where x represents the NA concentration and y represents the solvent used to treat the raw TMB-5 NA. For example, iPP/0.1TMB-5_LP_ means iPP containing 0.1 wt% TMB-5_LP_ NA.

A Linkam CSS-450 hot stage (Linkam Scientific Instruments, Ltd., Salfords, UK) was used to control the crystallization conditions. The iPP/TMB-5 blends were individually heated to 200 °C and kept for 10 min removing the mechanical and thermal history, then cooled to the set temperature for isothermal crystallization. The heating/cooling rate was 30°/min. The temperature set in the experiment included the crystallization temperature T_c_ and the pre-crystallization temperature T_pc_.

### 2.3. Measurement and Characterization

The crystalline structures of iPP and iPP/TMB-5 specimens were investigated via wide angle X-ray diffraction (WAXD) measurements, which were conducted by synchrotron radiation wide-angle X-ray diffraction at beamline 1W2A of the Beijing Synchrotron Radiation Facility (Beijing, China) with a wavelength λ = 0.154 nm. A Mar165-CCD was employed for collection of 2D WAXD images. The sample-to-detector distance was 126 mm for the WAXD measurements. The 2D WAXD patterns were analysed with the fit2D software and transformed into linear WAXD profiles.

The melting and non-isothermal crystallization behaviors of iPP/TMB-5 samples were investigated via DSC measurements (TA Q-2000) under N_2_ atmosphere. Both the heating and cooling rates were 10 °C/min.

### 2.4. Determination of β-crystal Content in Crystallized iPP/TMB-5 Samples

The relative amount of *β*-crystal (K*_β_*) in the crystallized iPP/TMB-5 specimens was calculated according to the method of Turner-Jones et al. [27], which was further modified by Hsiao et al. [28]. The calculation equation is as follows.

(1) $$K_{\beta }=\frac{A_{\beta (300)}}{A_{\alpha (110)}+A_{\alpha (040)}+A_{\alpha (130)}+A_{\beta (300)}}$$

where A*_β_*_(300)_, A*_α_*_(110)_, A*_α_*_(040)_ and A*_α_*_(130)_ are the areas of *β*(300), *α*(110), *α*(040) and *α*(130) diffraction peaks in the WAXD profiles, respectively.

## 3. Results and Discussion

### 3.1. Effects of Crystallization Temperature on Crystalline Structure of Different TMB-5 Nucleated iPP

**Figure 1:**
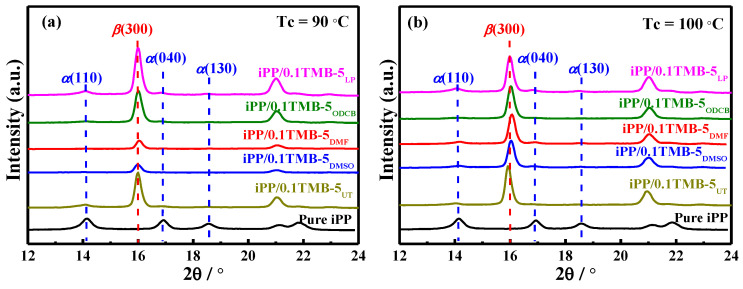

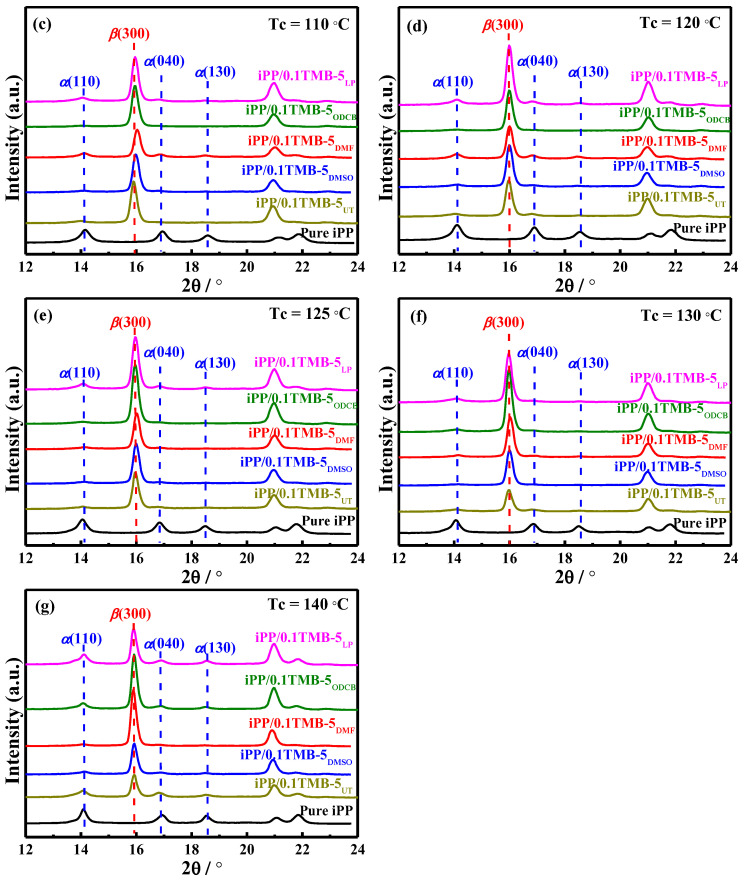
WAXD profiles of TMB-5 nucleated iPP (iPP/0.1TMB-5_UT_, iPP/0.1TMB-5_DMSO_, iPP/0.1TMB-5_DMF_, iPP/0.1TMB-5_ODCB_ and iPP/0.1TMB-5_LP_) and pure iPP which have statically crystallized at different crystallization temperatures: (**a**) T_c_ = 90 °C, (**b**) T_c_ = 100 °C, (**c**) T_c_ = 110 °C, (**d**) T_c_ = 120 °C, (**e**) T_c_ = 125 °C, (**f**) T_c_ = 130 °C, (**g**) T_c_ = 140 °C, the NA content is 0.1 wt%.

TMB-5 has been reported as a dual-selective *β*-nucleating agent for iPP which can induce the formation of both *α*-iPP and *β*-iPP depending on the thermal conditions [23]. It has been reported that TMB-5 can only induce *β*-iPP at crystallization temperature T_c_ = 135 °C and *α*-iPP at T_c_ = 145 °C, respectively [29]. Herein, we investigated the crystalline structure of iPP containing 0.1 wt% different TMB-5 NAs at different T_c_s, the WAXD results are shown in Figure 1. The T_c_s set in the present experiment was 90 °C, 100 °C, 110 °C, 120 °C, 125 °C, 130 °C and 140 °C. The crystallization time was set as 6 h to ensure full crystallization of the samples. The *β*(300), *α*(110), *α*(040) and *α*(130) diffraction peaks involved in Equation (1) are marked in the WAXD profiles. For comparison, WAXD profiles of pure iPP individually crystallized at 90 °C, 100 °C, 110 °C, 120 °C, 125 °C, 130 °C and 140 °C are also presented in Figure 1a–g. It is found that with the addition of 0.1 wt% TMB-5 NAs, the intensity of characteristic peaks of *α*-iPP crystal at 2θ = 14.1° (*α*(110)), 2θ = 16.8° (*α*(040)) and 2θ = 18.4° (*α*(130)) tremendously decreased while the intensity of the characteristic *β*(300) peak at 2θ = 16.0° distinctly increased (compared the WAXD profile of pure iPP with that of iPP/0.1TMB-5_UT_, iPP/0.1TMB-5_DMSO_, iPP/0.1TMB-5_DMF_, iPP/0.1TMB-5_ODCB_, and iPP/0.1TMB-5_LP_ in Figure 1a–g, respectively).

**Figure 2:**
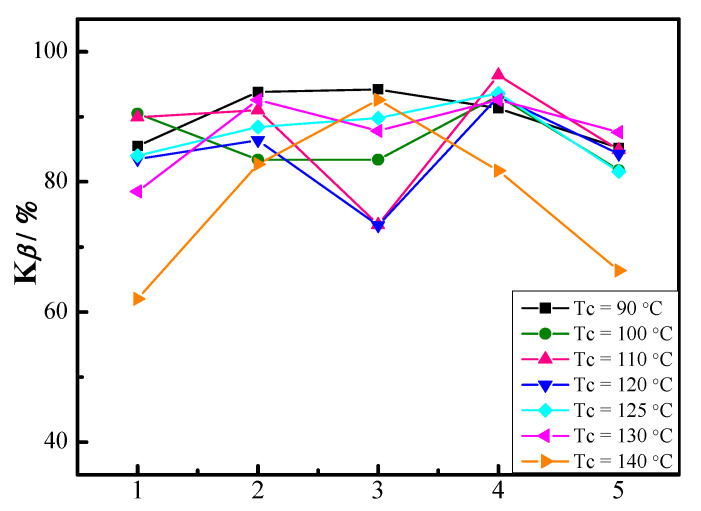
K*_β_* values of TMB-5 nucleated iPP statically crystallized at each individual crystallization temperatures, the NA content is 0.1 wt%. The horizontal axis numbers 1–5 correspond to iPP/0.1TMB-5_UT_, iPP/0.1TMB-5_DMSO_, iPP/0.1TMB-5_DMF_, iPP/0.1TMB-5_ODCB_ and iPP/0.1TMB-5_LP_, respectively.

**Figure 3:**
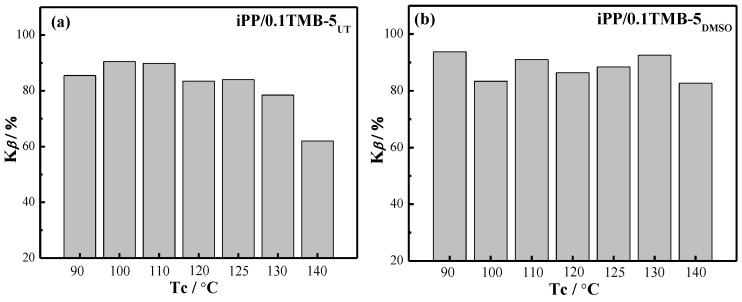

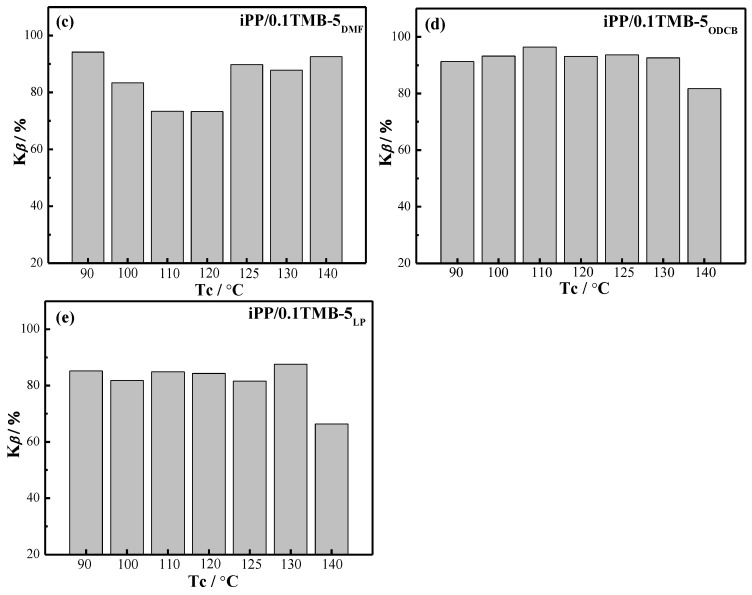
K*_β_* values of different TMB-5 nucleated iPP specimens: (**a**) iPP/0.1TMB-5_UT_, (**b**) iPP/0.1TMB-5_DMSO_, (**c**) iPP/0.1TMB-5_DMF_, (**d**) iPP/0.1TMB-5_ODCB_ and (**e**) iPP/0.1TMB-5_LP_ which have statically crystallized at different temperatures, the NA content is 0.1 wt%.

**Figure 4:**
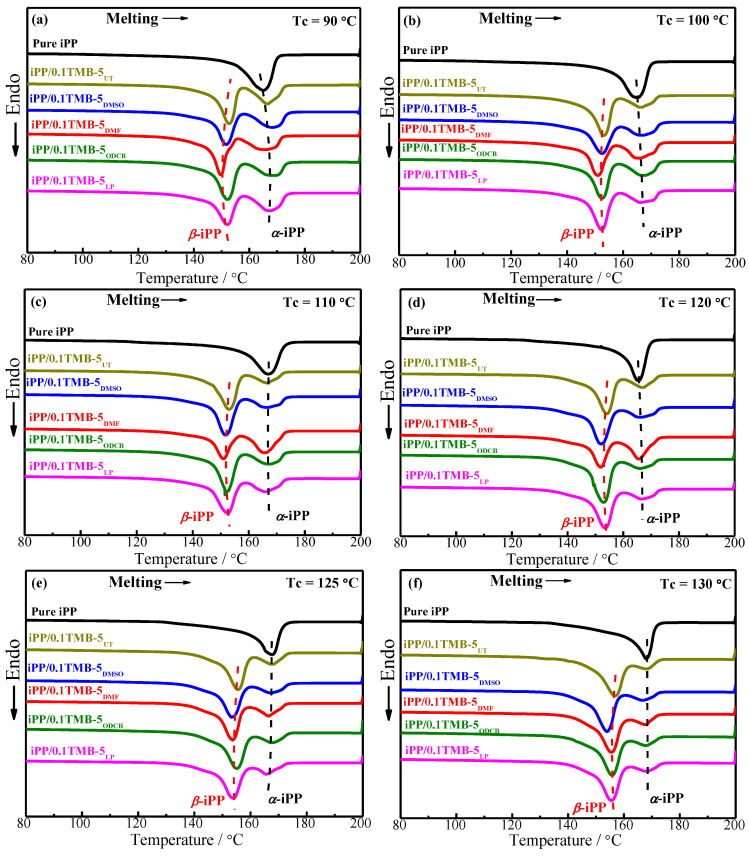

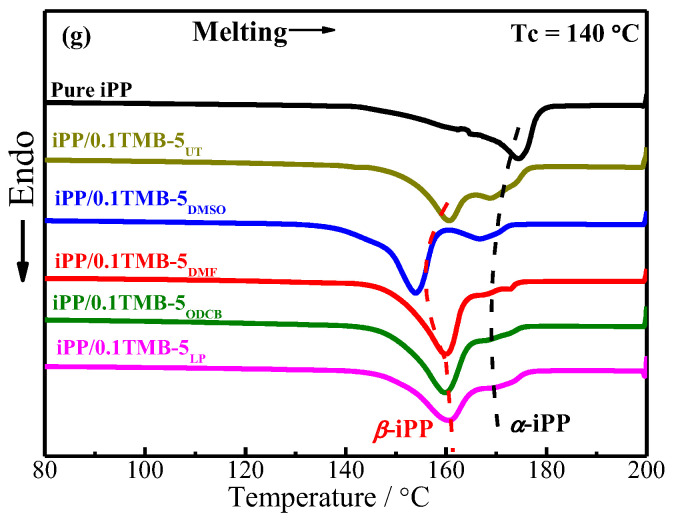
Effects of different solvent-treated TMB-5 NAs on the melting behaviors of iPP and iPP/TMB-5. All the iPP and iPP/TMB-5 specimens were already statically crystallized at each individual crystallization temperatures: (**a**) T_c_ = 90 °C, (**b**) T_c_ = 100 °C, (**c**) T_c_ = 110 °C, (**d**) T_c_ = 120 °C, (**e**) T_c_ = 125 °C, (**f**) T_c_ = 130 °C, (**g**) T_c_ = 140 °C, the NA content is 0.1 wt%, the heating rate is 10 °C/min.

The calculated K*_β_* values of iPP/0.1TMB-5_UT_, iPP/0.1TMB-5_DMSO_, iPP/0.1TMB-5_DMF_, iPP/0.1TMB-5_ODCB_, and iPP/0.1TMB-5_LP_ crystallized at each individual crystallization temperatures are shown in Figure 2. The results show that at each individual T_c_, the K*_β_* value varies among different TMB-5 nucleated iPP. It also indicates that when the crystallization temperature is below 140 °C the K*_β_* values of all iPP/0.1TMB-5 samples are almost above 80%. In addition, when crystallized at 140 °C (see the orange line in Figure 2), only iPP/0.1TMB-5_DMF_ exhibits a *β*-crystal fraction far above 80%. Further analysis reveals that (1) iPP/0.1TMB-5_DMSO_ (K*_β_* = 93.8%) and iPP/0.1TMB-5_DMF_ (K*_β_* = 94.2%) exhibit the highest K*_β_* values at T_c_ = 90 °C (see the black line in Figure 2); (2) iPP/0.1TMB-5_ODCB_ exhibits the highest K*_β_* values at T_c_ = 100 °C (K*_β_* = 93.2%), T_c_ = 110 °C (K*_β_* = 96.4%), T_c_ = 120 °C (K*_β_* = 93.1%) and T_c_ = 125 °C (K*_β_* = 93.6%) (see the green, pink, dark blue and light blue lines in Figure 2); (3) iPP/0.1TMB-5_DMSO_ (K*_β_* = 92.6%) and iPP/0.1TMB-5_ODCB_ (K*_β_* = 92.6%) exhibit the highest K*_β_* values at T_c_ = 130 °C (see the magenta line in Figure 2); (4) iPP/0.1TMB-5_DMF_ (K*_β_* = 92.6%) exhibit the highest K*_β_* value at T_c_ = 140 °C (see the orange line in Figure 2).

The temperature-dependence of K*_β_* value for each iPP/TMB-5 sample is shown in Figure 3. The results in Figure 3b,d indicate that the *β*-crystal fraction in TMB-5_DMSO_ and TMB-5_ODCB_ modified iPP is substantially unaffected by crystallization temperature. Under the studied crystallization temperature range 90–140 °C, K*_β_* values of iPP/TMB-5_DMSO_ keep in the range of 83–94% and iPP/TMB-5_ODCB_ keep in the range of 82–96.4%. Furthermore, all the K*_β_* values of iPP/TMB-5_ODCB_ are above 91.3% within a broad temperature range of 90–130 °C. Figure 3a,e indicate that the *β*-crystal inducing efficiency of TMB-5_UT_ and TMB-5_LP_ is seriously affected by high crystallization temperature, exhibiting an obvious decreased K*_β_* value from an average 80% at T_c_ = 90–130 °C to about 60% at T_c_ = 140 °C. Figure 3c reveals that the *β*-crystal inducing efficiency of TMB-5_DMF_ is very sensitive to crystallization temperature, exhibiting a K*_β_* value of ~90% at T_c_ = 90 °C and T_c_ = 125–140 °C, while 70–80% at T_c_ = 100–120 °C.

From Figure 2 and Figure 3, one can see that despite of the same chemical composition, TMB-5 treated by different solvent exhibit different *β*-crystal inducing efficiency and temperature-dependence. The ODCB-treated TMB-5 NA (TMB-5_ODCB_) exhibits a steady and high *β*-crystal inducing efficiency with K*_β_* = 93–96.4% within a broad temperature range T_c_ = 100–130 °C. The DMSO-treated TMB-5 NA (TMB-5_DMSO_) also exhibits a steady and relatively high *β*-crystal inducing efficiency with K*_β_* = 83–94% within the studied temperature range T_c_ = 90–140 °C. The DMF-treated TMB-5 NA is the most temperature-sensitive one, but it can induce a high fraction of *β*-crystal both at low temperature T_c_ = 90 °C (K*_β_* = 94.2%) and at high temperature T_c_ = 140 °C (K*_β_* = 92.6%). The untreated TMB-5 NA (TMB-5_UT_) and the LP-treated TMB-5 NA (TMB-5_LP_) exhibit a relatively steady *β*-crystal inducing efficiency within T_c_ = 90–130 °C with K*_β_* value around 80%.

Considering the polarity sequence of the solvents (DMSO > DMF > ODCB > LP), it can be concluded that the strong polar solvent treated TMB-5 NAs (such as TMB-5_DMSO_ and TMB-5_DMF_) induce more content of *β*-iPP crystal at high crystallization temperature (such as T_c_ = 140 °C) than the untreated TMB-5 (TMB-5_UT_) and the weak polar/non-polar solvent treated TMB-5 NAs (TMB-5_ODCB_/TMB-5_LP_).

Due to the better toughness, improved heat resistance [1], high impact and tear strength [7] and improved elongation at break [8] of *β*-iPP crystal, the above findings can be used to guide the production process, that is, to select the appropriate TMB-5 NA and the corresponding processing temperature according to the performance requirements of iPP products or to tailor the crystalline structure and properties of iPP products on purpose.

### 3.2. Melting and Non-Isothermal Crystallization Behaviors of Different iPP/TMB-5

Figure 4 shows the DSC melting curves of the already-crystallized pure iPP and iPP nucleated with five different solvent-treated TMB-5 NAs. According to Figure 4a–g, one can see that within the studied T_c_ range 90–140 °C, only the melting peak of *α*-crystal is shown for pure iPP. The main reason is that pure iPP is unlikely to form metastable *β*-iPP in the absence of external conditions [22]. It also can be seen that with addition of 0.1 wt% TMB-5 NA, melting peak of *β*-iPP crystal occurs with a sharpened shape while melting peak of *α*-iPP crystal significantly weakens and broadens. The result indicates that the addition of 0.1 wt% TMB-5 NA tremendously enhances the formation of *β*-iPP crystal within T_c_ = 90–140 °C, which is agreed with the WAXD results in Figure 1.

**Figure 5:**
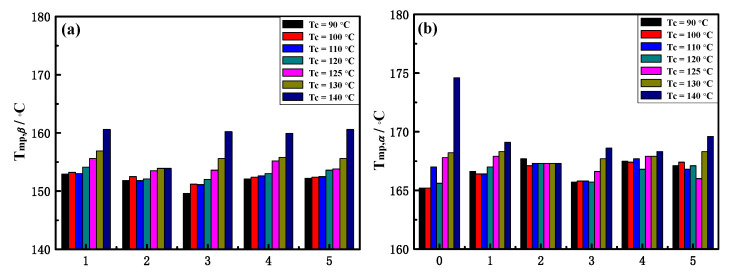
The peak melting temperatures (**a**) T_mp,*β*_ and (**b**) _Tmp,*α*_. The horizontal axis numbers 0–5 correspond to pure iPP, iPP/0.1TMB-5_UT_, iPP/0.1TMB-5_DMSO_, iPP/0.1TMB-5_DMF_, iPP/0.1TMB-5_ODCB_ and iPP/0.1TMB-5_LP_, respectively.

Notably, there is a difference in melting temperatures of *α*-crystal and *β*-crystal between pure iPP and the five different iPP/TMB-5 specimens. The peak melting temperature of *α*-crystal (T_mp,*α*_) and *β*-crystal (T_mp,*β*_) are shown in Figure 5. The horizontal axis numbers 0~5 in Figure 5 represent pure iPP, iPP/0.1TMB-5_UT_, iPP/0.1TMB-5_DMSO_, iPP/0.1TMB-5_DMF_, iPP/0.1TMB-5_ODCB_ and iPP/0.1TMB-5_LP_, respectively. Figure 5a shows the T_mp,*β*_ results, and Figure 5b shows the T_mp,*α*_ results. The same color of the column in Figure 5 means the same crystallization temperature. Comparing the black columns (T_c_ = 90 °C) in Figure 5b, one can see that the iPP/TMB-5 specimens (corresponding with horizontal axis numbers 1–5) exhibit higher T_mp,*α*_ than pure iPP (corresponding with horizontal axis number 0). This is also the case when T_c_ = 100 °C (comparing the red columns in Figure 5b). The higher melting temperature of *α*-crystal in iPP/TMB-5 specimens than pure iPP might be caused by the partial melting of metastable *β*-crystal which then recrystallized to form the more stable *α*-crystal during the DSC heating process [12]. This phenomenon is most evident at T_c_ = 90 °C and T_c_ = 100 °C, which indicates that with relatively low crystallization temperature the TMB-5 induced *β*-crystal has less perfect structure and less stability. The conclusion is consistent with the results in Figure 5a, that is, the T_mp,*β*_s of the five iPP/TMB-5 specimens are obviously lower at T_c_ = 90 °C (black columns) and T_c_ = 100 °C (red columns).

**Figure 6:**
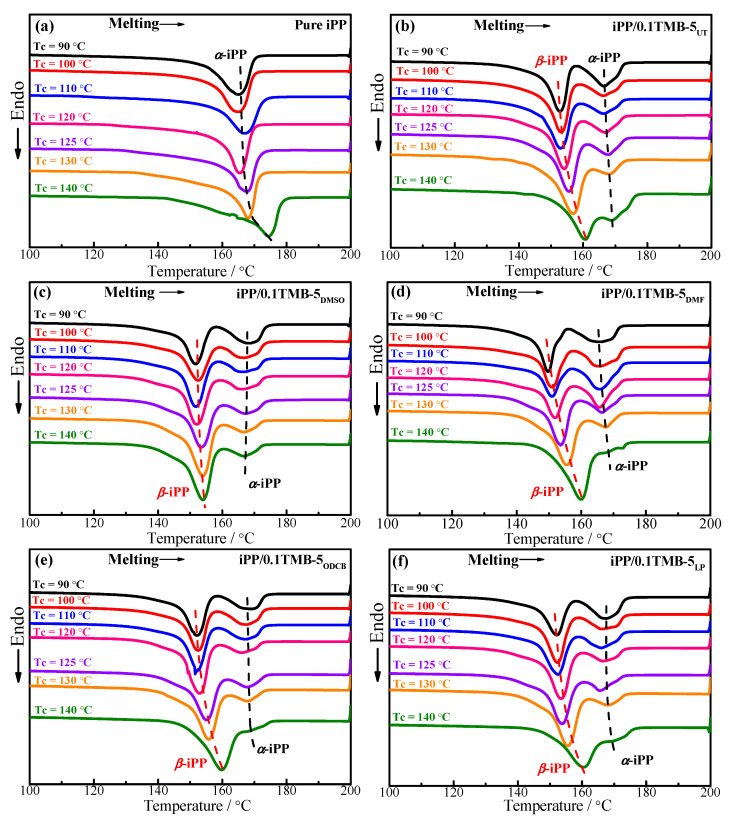
Dependence of melting behaviors of crystallized (**a**) pure iPP, (**b**) iPP/0.1TMB-5_UT_, (**c**) iPP/0.1TMB-5_DMSO_, (**d**) iPP/0.1TMB-5_DMF_, (**e**) iPP/0.1TMB-5_ODCB_ and (**f**) iPP/0.1TMB-5_LP_ on the crystallization temperature, the NA content is 0.1 wt%, the heating rate is 10 °C/min.

According to the dashed lines marked in Figure 4c–f, as well as the T_mp,*α*_ results in Figure 5b, it can be seen that, with increasing T_c_ (110–130 °C), the difference of T_mp,*α*_ between pure iPP and the five iPP/TMB-5 specimens are nearly eliminated, indicating less *β*-*α* transformation/recrystallization in iPP/TMB-5 occurred during DSC heating process. In other words, more stable and perfect *β*-crystal can be obtained at relatively high T_c_, such as 110–130 °C herein. Next, we investigated the dependence of melting behaviors of pure iPP and different TMB-5 nucleated iPP on the crystallization temperature. The DSC melting curves are shown in Figure 6.

Comparing the column height of each separate set of columns in Figure 5, it can be observed that the peak melting temperatures T_mp,*α*_ and T_mp,*β*_ increase with the crystallization temperature, except for iPP/TMB-5_DMSO_. This is consistent with the variation trends of dashed line marked in Figure 6. On one hand, the results indicate that appropriate increase of crystallization temperature is beneficial to improve the perfection degree of crystal structures, on the other hand, it indicates that different TMB-5 NAs exhibit different dependence on crystallization temperature when inducing the crystallization of iPP. The substantially unchanged T_mp,*α*_ and T_mp,*β*_ of iPP/0.1TMB-5_DMSO_ (see column groups with number 2 in Figure 5a,b) implies a relatively high consistency of iPP crystalline structure induced by TMB-5_DMSO_ within T_c_ = 90–140 °C. This is agreed with the results in Figure 3b that the *β*-crystal fraction of iPP/0.1TMB-5_DMSO_ keeps in a steady level of 83–94% within T_c_ = 90–140 °C. It is noteworthy that DMSO has the strongest polarity among all solvents used to treat TMB-5.

**Figure 7:**
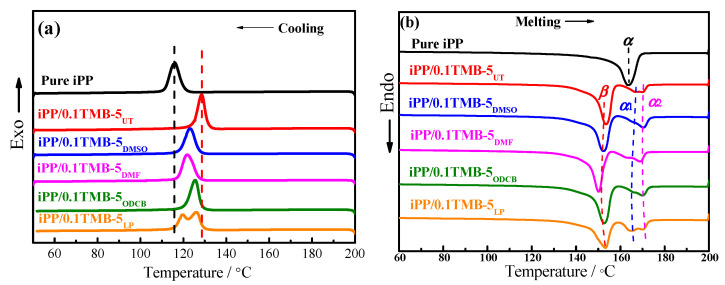
(**a**) DSC cooling curves after a 200 °C heating of 5 min to erase the thermal and mechanical history and (**b**) the subsequent melting curves of pure iPP and TMB-5 nucleated iPP (iPP/0.1TMB-5_UT_, iPP/0.1TMB-5_DMSO_, iPP/0.1TMB-5_DMF_, iPP/0.1TMB-5_ODCB_ and iPP/0.1TMB-5_LP_). The NA content is 0.1 wt%, both the heating and cooling rate are 10 °C/min.

The non-isothermal crystallization and the subsequent melting behaviors of pure iPP and different TMB-5 nucleated iPP were investigated via DSC with the heating/cooling rate of 10 °C/min. The crystallization and melting curves are shown in Figure 7. It can be observed from Figure 7a that the crystallization peak is evidently shifted to higher temperature with the addition of 0.1 wt% TMB-5 NAs. From the corresponding melting curves in Figure 7b, a single melting peak around 165 °C is found for pure iPP. With the addition of 0.1 wt% TMB-5 NAs, sharpened melting peaks around 155 °C occur indicative of the generation of *β*-crystal. Melting peaks corresponding to *α*-crystal also occur for the TMB-5 nucleated iPP, with weaker peak intensity and a widened shape. As can be observed from Figure 7b, all the DSC melting curves of the five TMB-5 nucleated iPP specimens show two *α*-crystal melting peaks. For the convenience of analysis, the onset crystallization temperature T_c,onset_, the crystallization peak temperature T_cp_, the peak melting temperature of *α*-crystal (T_mp,*α*_, T_mp,*α*1_ and T_mp,*α*2_) and the peak melting temperature of *β*-crystal (T_mp,*β*_) corresponding to Figure 7 are listed in Table 1.

The *α*-melting peak at lower temperature around 163–166 °C represents *α*_1_-phase with less structure perfection and stability, while the peak at higher temperature around 170 °C represents *α*_2_-phase with higher structure perfection and stability. The *α*-crystal might come from two aspects: first, the crystallization of *α*-crystal during the previous DSC cooling procedure when considering the dual nucleating ability of TMB-5 [29,30], second, the *β*-*α* recrystallization during DSC heating process [24,26,31]. A similar explanation can be used to interpret the occurrence of *α*_2_-phase, that is, the less perfect or less stable *α*_1_-phase melted and recrystallized into the more stable *α*_2_-phase during DSC heating process. It can also be observed that T_mp,*α*_s (both T_mp,*α*1_ and T_mp,*α*2_) of all iPP/0.1TMB-5 specimens are higher than the T_mp,*α*_ of pure iPP, which indicates that TMB-5 NAs can not only induce the formation of *β*-iPP crystal, but also enhance the stability of *α*-iPP crystal.

From Figure 7b and Table 1, we can find that the *β*-crystal melting peak width of iPP/0.1TMB-5_UT_ is the narrowest and the T_mp,*β*_ of iPP/0.1TMB-5_UT_ is relatively higher than the others. It indicates that the TMB-5_UT_ induced *β*-crystals formed via the non-isothermal crystallization possess a relatively higher structure perfection and stability. On the other hand, iPP/0.1TMB-5_LP_ exhibits the widest *β*-crystal melting peak (see the orange curve in Figure 7b), implying the crystalline structure of *β*-crystal induced by TMB-5_LP_ is less perfect. According to the melting curve of iPP/0.1TMB-5_LP_ in Figure 7b, it can be observed that the area of *β*-crystal melting peak is close to *α*-crystal melting peak, implying a competitive formation of *α*-crystal in iPP/0.1TMB-5_LP_ during the non-isothermal crystallization process. Furthermore, the similar intensity of iPP/0.1TMB-5_LP_ *α*_1_-phase and *α*_2_-phase melting peaks is obviously different from other iPP/0.1TMB-5 specimens, implying less *α*_1_-*α*_2_ recrystallization during the heating process. Combing the above analyses, the *α*-crystal in iPP/TMB-5_LP_ is more likely to have formed with considerable quantity and stability during the non-isothermal crystallization process. This explains the abnormal double crystallization peaks of iPP/0.1TMB-5_LP_ in Figure 7a.

In addition, we use the value of T_c,onset_-T_cp_ to evaluate the crystallization rate of pure iPP and iPP/0.1TMB-5 during the non-isothermal crystallization process, that is the smaller T_c,onset_-T_cp_ value, the faster crystallization rate. According to Table 1, T_c,onset_-T_cp_ values of the five iPP/0.1TMB-5 specimens are smaller than that of pure iPP, implying that TMB-5 essentially act as an effective nucleating agent for iPP and enhances the crystallization rate of iPP during non-isothermal crystallization. Furthermore, the T_c,onset_-T_cp_ values of iPP/0.1TMB-5_UT_ and iPP/0.1TMB-5_LP_ are 4.7 °C and 4.6 °C, respectively, which are smaller than that of iPP/0.1TMB-5_DMSO_ (6.0 °C), iPP/0.1TMB-5_DMF_ (6.1 °C) and iPP/0.1TMB-5_ODCB_ (5.2 °C). The results indicate that the untreated TMB-5 (TMB-5_UT_) and non-polar solvent treated TMB-5 (TMB-5_LP_) exhibit higher nucleating efficiency than the polar solvent treated TMB-5 NAs (TMB-5_DMSO_, TMB-5_DMF_ and TMB-5_ODCB_) during the non-isothermal crystallization of iPP.

### 3.3. Effects of High-Temperature Pre-Crystallization on Crystalline Structure of Different TMB-5 Nucleated iPP

**Figure 8:**
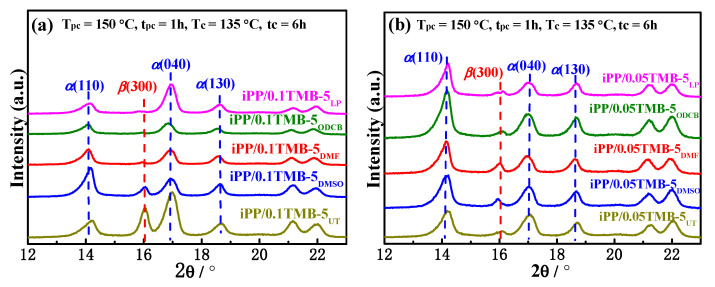
WAXD profiles of different TMB-5 nucleated iPP statically crystallized at T_c_ = 135 °C for 6 h after a pre-crystallization at T_pc_ = 150 °C for 1 h, the NA content is (**a**) 0.1 wt% and (**b**) 0.05 wt%.

As reported, TMB-5 is a dual-selective nucleating agent for iPP depending on the thermal conditions [23,24,29,30]. In our previous work [32], it was found that high-temperature crystallization at above 145 °C tremendously inhibit the formation of *β*-crystal in iPP containing 0.1 wt% TMB-5. In present work, we used different solvent-treated TMB-5 as the *β*-NA for iPP. A high-temperature pre-crystallization at 150 °C (T_pc_ = 150 °C) for 1 h (t_pc_ = 1 h) was introduced before the isothermal crystallization at 135 °C (T_c_ = 135 °C) for 6 h (t_c_ = 6 h). The TMB-5 concentration was 0.1 wt% and 0.05 wt%, and the corresponding WAXD results are shown in Figure 8a and Figure 8b, respectively.

**Figure 9:**
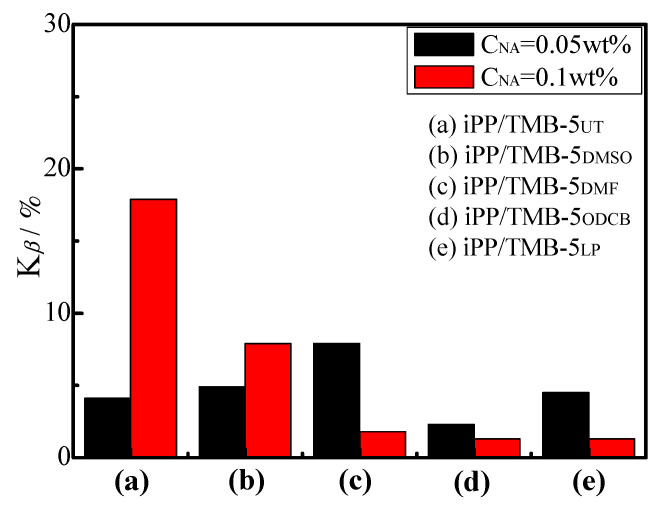
K*_β_* values of different TMB-5 nucleated iPP specimens: (**a**) iPP/TMB-5_UT_, (**b**) iPP/TMB-5_DMSO_, (**c**) iPP/TMB-5_DMF_, (**d**) iPP/TMB-5_ODCB_ and (**e**) iPP/TMB-5_LP_ which statically crystallized at T_c_ = 135 °C for 6 h after a pre-crystallization at T_pc_ = 150 °C for 1 h, the NA content is 0.1 wt% (red column) and 0.05 wt% (black column).

The representative diffraction peaks of *α*-crystal (*α*(110), *α*(040) and *α*(130)) and *β*-crystal (*β*(300)) are marked in Figure 8. Comparing the WAXD profiles of Figure 8a with Figure 1, one can see that the intensities of *β*(300) peaks are tremendously decreased and the *α*-peaks are predominant, indicative of a serious reduction in *β*-crystal in the 150 °C pre-crystallized iPP/0.1TMB-5 specimens. A similar phenomenon is also observed for iPP containing 0.05 wt% TMB-5 NAs (see Figure 8b). The corresponding K*_β_* values calculated via Equation (1) are shown in Figure 9. The black columns and red columns correspond to iPP/0.05TMB-5 and iPP/0.1TMB-5 specimens, respectively. It is obvious that the K*_β_* values of iPP/TMB-5 with a 150 °C pre-crystallization are far less than those isothermally crystallized at 90–140 °C (comparing results in Figure 3 and Figure 9).

Comparing the red columns in Figure 9, it can be observed that with the NA concentration of 0.1 wt%, the untreated TMB-5 (TMB-5_UT_) induced more *β*-crystal than the solvent treated TMB-5 NAs. Furthermore, TMB-5 treated by strong polar solvent (TMB-5_DMSO_) induced more *β*-crystal than those treated by less polar solvent (TMB-5_DMF_ and TMB-5_ODCB_) and non-polar solvent (TMB-5_LP_). As reported in our previous work, TMB-5 induced iPP to form *α*-crystal instead of *β*-crystal at 150 °C, in other words, the pre-formed *α*-crystal induced by TMB-5 at 150 °C inhibits the *β*-crystal inducing ability of TMB-5 [32]. Thus, the higher *β*-crystal fraction of iPP/0.1TMB-5_UT_ is considered to result from the less formation of TMB-5_UT_ induced *α*-crystal at high temperature (T_pc_ = 150 °C). It can also be inferred that *α*-crystals are tremendously formed at 150 °C in iPP/0.1TMB-5_DMSO_, iPP/0.1TMB-5_DMF_, iPP/0.1TMB-5_ODCB_ and iPP/0.1TMB-5_LP_, thus inhibiting the formation of *β*-crystal.

Comparing the black columns with the red columns in Figure 9, it can be observed that K*_β_* values of iPP/0.1TMB-5_UT_ and iPP/0.1TMB-5_DMSO_ are, respectively, larger than that of iPP/0.05TMB-5_UT_ and iPP/0.05TMB-5_DMSO_, while K*_β_* values of iPP/0.1TMB-5_DMF_, iPP/0.1TMB-5_ODCB_ and iPP/0.1TMB-5_LP_ are, respectively, smaller than that of iPP/0.05TMB-5_DMF_, iPP/0.05TMB-5_ODCB_ and iPP/0.05TMB-5_LP_. It can be explained that with the addition of 0.1 wt% TMB-5_DMF_, 0.1 wt% TMB-5_ODCB_ and 0.1 wt% iPP/0.1TMB-5_LP_, large amounts of *α*-crystals would be induced during the 150 °C pre-crystallization of iPP, tremendously restraining the generation of *β*-crystal. On the contrary, less *α*-crystals would be induced to form during 150 °C pre-crystallization with less loading of TMB-5_DMF_, TMB-5_ODCB_ and TMB-5_LP_ (C_NA_ = 0.05 wt%), thus reducing the restriction on *β*-crystal formation to some extent. However, as shown in Figure 9, most of the K*_β_* values are less than 10%, indicating that no matter what kind of TMB-5 NA is used, high temperature pre-crystallization is not conducive to the formation of *β*-crystal.

## 4. Conclusions

In present work, the effects of temperature on the crystalline structure of iPP containing different TMB-5 NAs, as well as the crystallization and melting behaviors of different iPP/TMB-5 specimens were investigated. The TMB-5 NAs were previously treated with different solvents before blending with iPP matrix. WAXD results indicate that within the studied isothermal crystallization temperature range 90–140 °C, the addition of 0.1 wt% TMB-5 NAs tremendously promotes the formation of *β*-iPP crystal. The stronger polarity of the solvent used to treat TMB-5, the higher *β*-crystal fraction of the TMB-5 nucleated iPP at high T_c_ = 140 °C. The *β*-crystal inducing efficiency of untreated TMB-5 (TMB-5_UT_) and non-polar solvent treated TMB-5 (TMB-5_LP_) is seriously reduced at high T_c_ = 140 °C. TMB-5_DMSO_ and TMB-5_ODCB_ can induce iPP to form a high and stable content of *β*-crystal within T_c_ = 90–140 °C. In addition, iPP crystals induced by TMB-5_DMSO_ exhibit a relatively high structural consistency within T_c_ = 90–140 °C, and *β*-crystal induced by TMB-5_ODCB_ keeps with an outstanding high content that K*_β_* > 91.3% within a broad temperature range T_c_ = 90–130 °C. TMB-5_DMF_ is the most temperature-sensitive one, but can induce a high content of *β*-crystal with K*_β_* > 92% both at low T_c_ = 90 °C and high T_c_ = 140 °C. High temperature pre-crystallization at T_pc_ = 150 °C evidently inhibits the formation of *β*-crystal for all iPP/TMB-5 specimens, with the K*_β_* values dropping sharply to below 10%.

DSC melting results of the isothermally crystallized iPP/0.1TMB-5 specimens indicate that TMB-5 induced *β*-crystal at low T_c_s exhibits less structural perfection and less stability. Appropriate increase of T_c_ is in favor of the structural perfection and stability of *β*-crystal. Among the five different TMB-5 NAs, iPP crystals induced by TMB-5_DMSO_ possess a relatively high structural consistency within T_c_ = 90–140 °C. The non-isothermal crystallization and melting DSC results indicate that TMB-5 is an effective nucleating agent to improve the crystallization temperature and the crystallization rate of iPP. Among the five different TMB-5 NAs, the untreated TMB-5 (TMB-5_UT_) and non-polar solvent treated TMB-5 (TMB-5_LP_) exhibit higher nucleating efficiency than the polar solvent treated TMB-5 NAs (TMB-5_DMSO_, TMB-5_DMF_ and TMB-5_ODCB_). Furthermore, *β*-crystal induced by TMB-5_UT_ possesses higher structural perfection and stability than the other TMB-5 NAs, while TMB-5_LP_ is more likely to induce the formation of *α*-crystal with considerable quantity and stability during the non-isothermal crystallization process.

The findings in our present work can be used to guide the processing and production of iPP products, tailoring the crystalline structure and properties of iPP by selecting the appropriate TMB-5 NA and the corresponding temperature conditions. In the future, we will study how to achieve artificial control of nucleating agent morphology through solvent treatment and subsequently to quantitatively regulate the crystalline structure and properties of iPP.

## Figures and Tables

**Table 1:** Crystallization and melting parameters of pure iPP and iPP/0.1TMB-5 specimens from DSC curves.

Sample	T_c,onset_/°C	T_cp_/°C	T_c,onset_-T_cp_/°C	T_mp*,β*_/°C	T_mp*,α*_/°C	T_mp*,α*1_/°C	T_mp*,α*2_/°C
Pure iPP	122.9	115.8	7.1	---	163.6	---	---
iPP/0.1TMB-5_UT_	133.2	128.5	4.7	153.7	---	166.4	170.3
iPP/0.1TMB-5_DMSO_	129.1	123.1	6.0	152.5	---	165.2	170.7
iPP/0.1TMB-5_DMF_	128.3	122.2	6.1	150.3	---	162.6	169.0
iPP/0.1TMB-5_ODCB_	130.6	125.4	5.2	153.0	---	165.2	170.2
iPP/0.1TMB-5_LP_	130.6	126.0	4.6	153.4	---	164.3	170.3

## Data Availability

The data that support the findings of this study are available within this article.

## Supplementary Materials

The following supporting information can be downloaded at: https://www.mdpi.com/article/10.3390/polym15030514/s1, Figure S1: Optical microscopic pictures of the five solvent treated TMB-5 nucleating agents.
