# Supplementary material for: Temperature Effects on the Crystalline Structure of iPP Containing Different Solvent-Treated TMB-5 Nucleating Agents
Source: Polymers (Basel). 2023 Jan 18;15(3):514. doi: 10.3390/polym15030514 (PMC9919969; doi:10.3390/polym15030514)
Supplement: Supplementary file 1 [file polymers-15-00514-s001.zip › polymers-2164926-supplementary.pdf]

Supplementary Materials

Temperature Effects on the Crystalline Structure of iPP Containing Different Solvent-treated TMB-5 Nucleating Agents

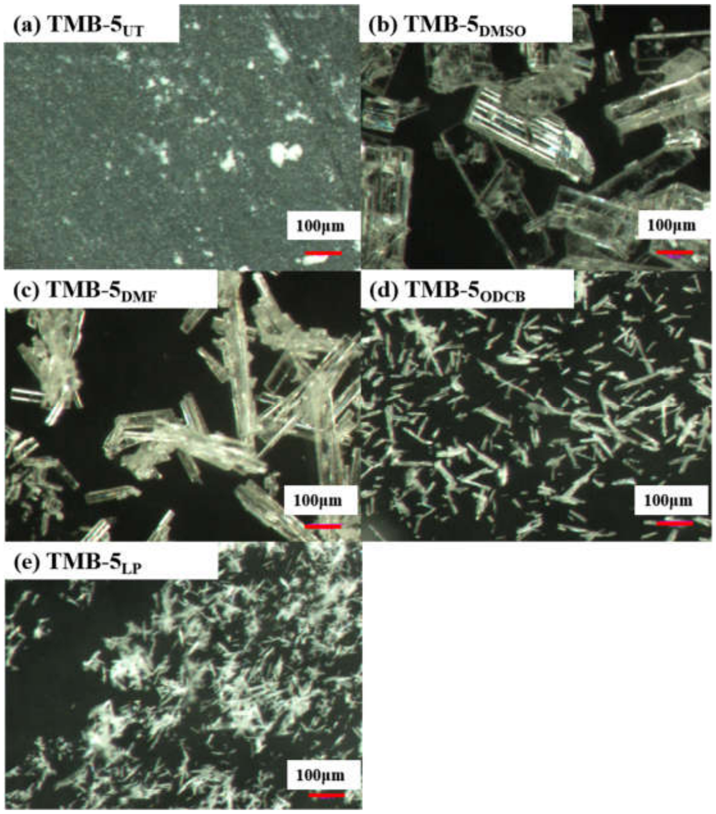

Figure S1. Optical microscopic pictures of the five solvent treated TMB-5 nucleating agents (NAs) which were used as the *β*-NA of iPP in present work. (**a**) untreated TMB-5 (TMB-5_{UT}), (**b**) dimethylsulfoxide treated TMB-5 (TMB-5_{DMSO}), (**c**) dimethyl formamide treated TMB-5 (TMB-5_{DMF}), (**d**) ortho-dichlorobenzene treated TMB-5 (TMB-5_{ODCB}), and (**e**) liquid paraffin treated TMB-5 (TMB-5_{LP}).
